# The effect of three different toe props on plantar pressure and patient comfort

**DOI:** 10.1186/1757-1146-5-22

**Published:** 2012-08-29

**Authors:** Sarah Johnson, Helen Branthwaite, Roozbeh Naemi, Nachiappan Chockalingam

**Affiliations:** 1 Centre for Sport, Health and Exercise Research, Faculty of Health, Staffordshire University, Stoke-On-Trent, UK

**Keywords:** Apical peak pressure, Digital deformities, Toe prop therapy, Comfort

## Abstract

**Background:**

Arthritic toe pathologies frequently lead to the development of painful apical pressure skin lesions that can compromise gait and affect quality of life. Historically conservative treatments involve the use of a toe prop with the intended aim of reducing plantar pressure from the apex of the digit. However, the effect of toe prop treatment on plantar digital pressure has not been investigated.

**Method:**

Twenty two subjects were recruited with lesser digital deformities and associated apical skin lesions. Individual pressure sensors were placed on the apices of the lesser toes and pressure was recorded under three toe prop conditions (leather, gel and silicone mould). A modified comfort index was utilised to assess the comfort of each condition.

**Results:**

Significant difference (*p* < 0.05) in mean peak pressure was observed at the apex of the 2^nd^ toe when using the gel (*p* < 0.001) and silicone (*p* < 0.001) toe prop compared to no toe prop. There was also a significant difference in the mean pressure time integral at the apex of the 2^nd^ toe when using gel (*p* < 0.001) and silicone (*p* < 0.004) toe props. There was no significant correlation between comfort and the recorded peak pressures. However, there was an indication that the silicone toe prop was more comfortable.

**Conclusion:**

As compared to the leather and silicone mould toe props, gel toe props were found to be the most effective for reducing peak pressure and pressure time integral on the apex of the second digit in patients with claw or hammer toe deformity.

## Background

Toes play an important role in dynamic foot function by increasing the weight bearing area of the forefoot. This increased area allows for sufficient plantar pressure to be exerted over a fixed point from which the body can then be propelled forward [1,2]. The digits stability and plantar flexion movements in a healthy foot that give this propulsion are controlled by the foot flexor muscles and plantar fascia arrangement [2-4].

The development of painful arthritic toe pathologies occurs over a long period of time, where alterations in propulsion can change joint positions. This can lead to pressure related skin lesions that compromise gait and can affect quality of life [5]. Digital deformities in the sagittal plane include claw, hammer, retracted and mallet toes. These digital deformities are diagnosed depending on which digital joint is maintained in a flexed or extended position. A claw toe is a flexus digitus deformity involving hyperextension of the metatarso-phalangeal joint (MTP) combined with flexion of the distal and proximal interphalangeal joints (IP) [6]. A hammered toe involves hyperextension of the MTP Joint and a flexion deformity of the proximal IP Joint, however there is no flexion deformity at the distal IP joint [6-8].

Clawed and hammered digits are more frequently associated with apical lesions particularly in the second digit which can bear 25% of digital peak plantar pressure and can be a main source of discomfort in the forefoot [1,9-11]. In these digital deformities the apex of the toe comes into contact with the ground first rather than the plantar fat pad that protects the distal phalanx. This increases the apical plantar pressure by reducing the contact area of the digit [8]. Retracted and mallet digital deformities do not present with such increased plantar pressure and apical lesions are therefore less common in these two type of deformities.

Skin lesions forming on the apex of the digit are a result of hyperkeratosis (locally increased rate of proliferation of keratinocytes), a normal physiologic response of the skin to chronic excessive pressure [12]. If pressure and tissue stress continue over time without symptom relief then the tissue can ulcerate and in some cases this can lead to toe amputation [13,14]. Off-loading pressure from the apex of the digit provides an indispensable precondition both for encouraging the tissue-repair mechanism, where active lesions are present, and also for stopping the potential progression of pre-ulcerative conditions toward lesions [12].

Toe props have been used to treat painful apical skin lesions for over 80 years and are placed under the claw or hammer digit with an aim to decrease plantar digital pressure [15-18]. Different types of toe props used for the treatment of apical lesions vary extensively. In clinical practice the size of the toe prop fitted depends on the length of digit, severity of the clawing and the comfort of the prop. A toe prop which is too large or ill fitting may well reduce apical pressure, as the toe apex will not touch the ground, but increased bulk can restrict function of the digits, create dorsal lesions and be uncomfortable to wear. A correctly fitted toe prop (shape and size) is still likely to reduce apical pressure and coincide with a better functioning toe increasing the comfort of the prop.

To date there has been minimal research into the actual effect of the toe prop on apical pressure and the practice of apical lesion management with a toe prop is mainly based on tradition. A further understanding of pressure reduction, comfort and effect of different toe props will enhance clinical practice in the management of digital foot pain. The aim of this study is to investigate the effect of 3 toe props regularly used in clinical practice for the treatment of callus related to apical pressures in subjects who have been diagnosed with either claw or hammer digital deformities. Additionally, the comfort of the props used will also be assessed as the reduction in pressure with a toe prop is known clinically for not always being tolerated by the patient.

## Methods

Prior to commencing this study ethical approval was sought and granted by Staffordshire University Research Ethics Committee, Stoke-on-Trent, UK. All subjects recruited provided written informed consent prior to the participation in this study.

A repeated measures design was employed to allow comparisons between the three toe prop conditions. Twenty two subjects (18 female: 4 male. Age 68 SD +/− 10years. Weight 73.21 kg SD +/− 10.5 kg. Height 168.4 cm SD+/− 8.82cm.) were recruited from a clinical population. All subjects presented with clawing/hammer of all lesser digits (second third fourth and fifth). The frequency of prominent digital deformities over the other digits included; second *n* = 9, third *n* = 2, fourth *n* = 2 and fifth *n* = 3. Hyperkeratotic lesions were present in frequency on the apex of the second *n* = 12, third *n* = 11, fourth *n* = 1 and fifth *n* = 0. Subjects were excluded if they had no apical lesion, complex ulcerations, foot surgical intervention, inappropriate footwear and an inability to walk unaided.

### Subject preparation

One week prior to data collection all recruited subjects received debridement of the apical lesion. This allowed for all subjects to be relieved from any direct pain related to the apical lesion. There have been mixed results regarding callus removal with reports supporting and negating the benefit of callus removal on pressure [13,19]. To gain a comparable comfort score removal of callus was deemed appropriate. Digital deformity was recorded as either flexible or rigid at each of the proximal and distal interphalangeal joints. Footwear was not standardised to portray an appropriate everyday lifestyle condition. Subjects own footwear used in the study was assessed for depth, width and length. Subjects were excluded if the footwear was tight and influenced toe spacing and depth.

**Figure 1:**
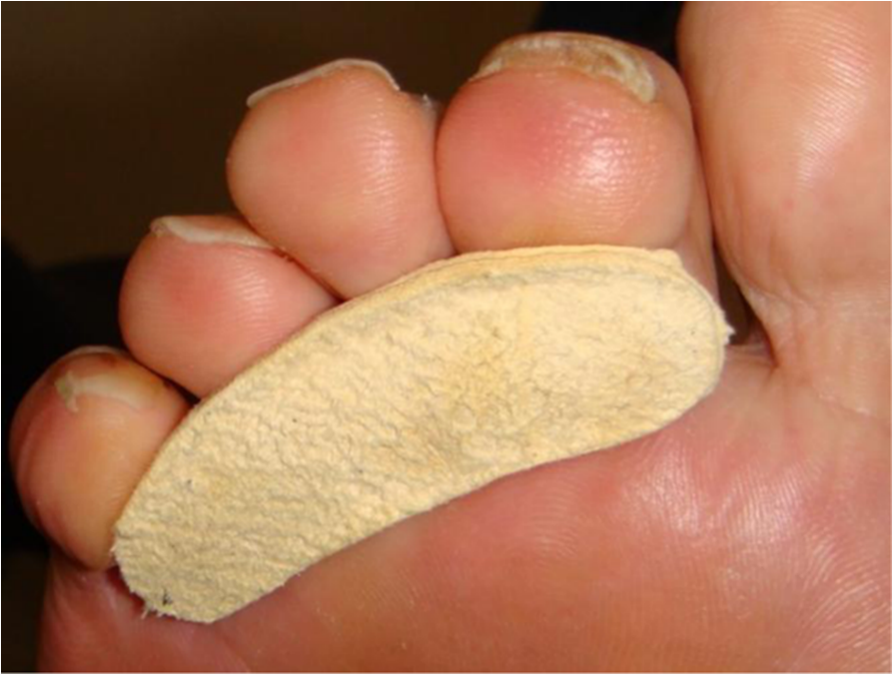
**Leather toe prop.** Leather toe props were fitted to digit size and were plantigrade with 3^rd^ metatarsal head.

**Figure 2:**
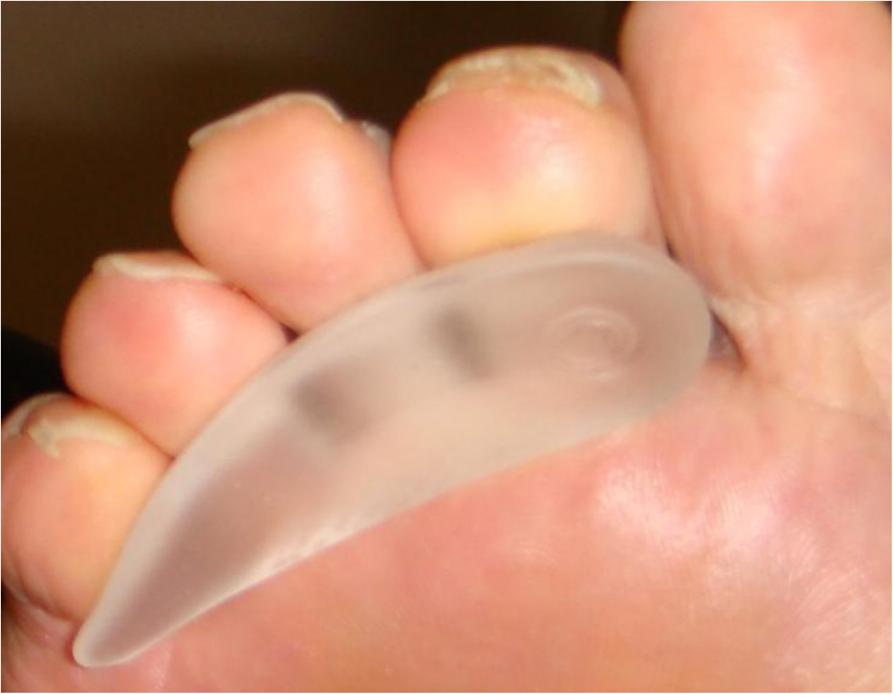
**Gel toe prop.** Gel toe props were similarly fitted to digit size and plantigrade with 3^rd^ metatarsal.

**Figure 3:**
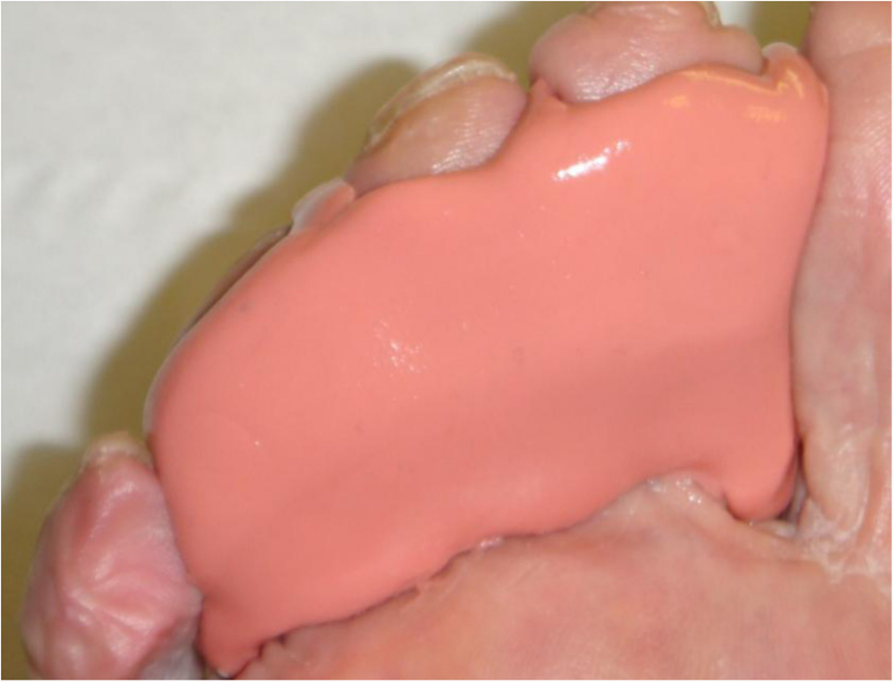
**Silicone toe prop.** Silicone toe props were individually molded to the contours of the digits.

All recruited subjects were fitted with three types of toe props to be researched (Figures 1, 2 and 3). Leather and gel toe props are preformed devices that are fitted based on foot and digit size. Silicone mould toe props are bespoke and individually molded to the digit. To keep within repeated measure design the prefabricated measured toe props (leather and gel) were assessed for fit by evaluating the position in relation to the plantigrade surface of the foot. A flush prop with the plantar third metatarsal head was classified as suitable, anything protruding or regressing from the plantar surface was refitted. Silicone moulds were manufactured for 2, 3, and 4 digits with the silicone spread evenly on to the apices of the distal phalanges with no added bulk under the proximal phalanges. The size, shape and form can vary with each patient depending on the participants toe structure therefore to minimize variance all toe props were dispensed by the same researcher.

### Data collection

A week after preparation, data were collected for each subject for each toe prop condition and a no toe prop condition (as control). Prior to testing all subjects were asked to complete a visual analogue scale, modified comfort index [20]. The scale evaluated the comfort of 10 parameters associated with the toe prop fit, size and feel and is based on a visual analogue scale of 10 cm. The line is a representation of how comfortable the toe prop is from 0 cm being very uncomfortable to 10 cm being very comfortable. A cumulative score from each parameter is generated and is an indication of overall comfort. A maximum score of 100 indicates extreme comfort.

Toe prop conditions were randomly allocated by the subject picking a card blinded to the researcher identifying the trial condition. Cards were continued to be picked until a full order of testing conditions was obtained. After each toe prop condition was applied to the digits the subjects were asked to walk around for 2 minutes and evaluate each condition for comfort. This was again collected using the comfort index.

**Figure 4:**
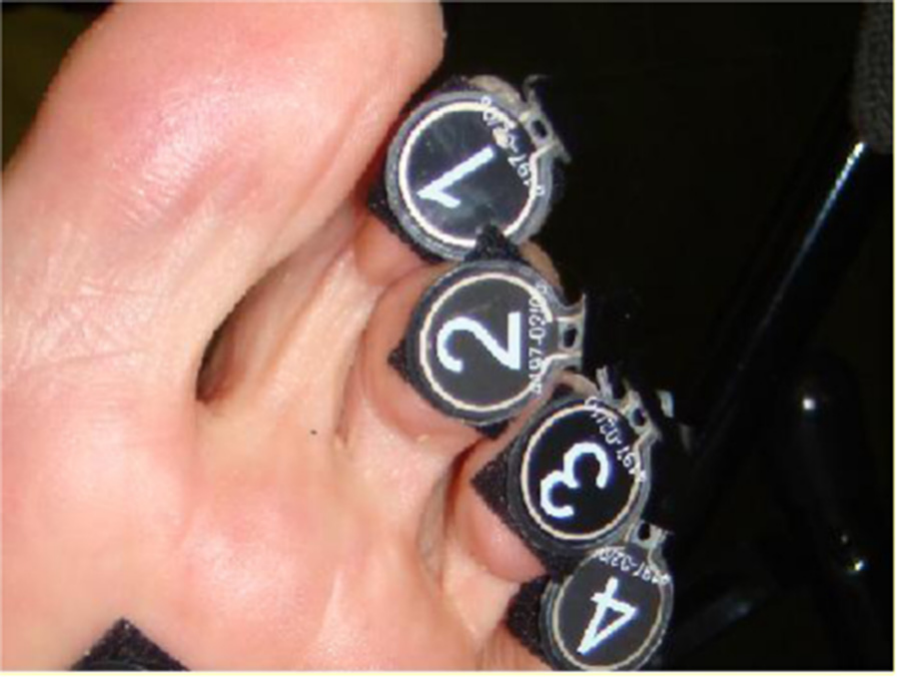
Sensor placement: 1 = Apex of second digit, 2 = Apex of third digit, 3 = Apex of fourth digit and 4 = Apex of fifth digit.

On completion of recording the comfort score, plantar pressure data were collected using Walkinsense (Tomorrow Options Microelectronics, S.A. Sheffield, UK). The sensors are individual piezoresistive force 100Hz sensors that were attached to the apices of each lesser toe 1–4 (Figure 4). The sensors are comparable to other plantar pressure systems but caution should be taken for a direct comparison of results between software [21]. The foot was cleaned and adhesive spray was applied to improve adhesion of the sensors. These sensors were connected to a wireless blue tooth device on the ankle, which is held in place by a Velcro strapping [21]. Proprietary software supplied by the manufacturers was set to receive data corresponding to the foot tested. Pressure data were collected whilst the subject walked at a self selected speed along a 10 meter walkway. From this data eight foot falls were recorded.

### Data analysis

Statistical analysis was conducted using SPSS (IBM v 19) software. Tests for normality to ensure the assumptions of parametric testing were met included the Kolmogorov-Smirnov statistic (>0.5 indicating normality). For data that met the assumptions parametric testing was implemented, for all data that did not meet the assumptions for normality non parametric equivalents were applied.

For each toe prop variable the comfort results were analysed using a non parametric Friedman test, alpha set at 0.05.

After removing the first and last steps, three foot falls with comparable pressure data were extracted, analysed and averaged [22]. The mean peak pressure and pressure time integral were calculated for each sensor in each of the four conditions. This data was statistically analysed using a one way repeated measure analysis (ANOVA) with alpha set at 0.05.

## Results

### Mean plantar digital peak pressure

**Figure 5:**
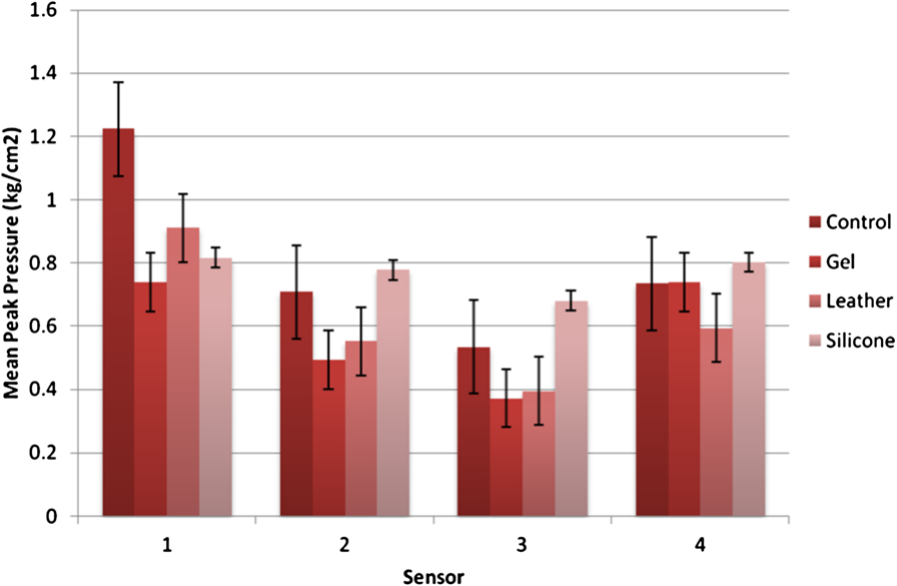
**Mean Peak pressure results.** Mean Peak Pressure for each sensor for each condition with error bars.

The mean plantar digital peak pressure for each sensor is reported in Figure 5. For all sensors the leather toe prop reduced peak pressure from the no toe prop condition. The gel toe prop also reduced mean peak plantar digital pressure for all sensors accept sensor 4. The silicone toe prop reduced mean peak plantar digital pressure compared to no toe prop condition for sensor 1 only, sensor 2,3 and 4 recorded higher mean peak plantar digital pressure. Significant difference in mean peak apical pressure at the apex of the 2^nd^ toe (sensor 1) (Wilks’ Lambda = .27, *F* (3,19) = 16.38, *p* < 0.001, multivariate partial eta squared = .72) was observed. Significance was seen when introducing the gel and silicone toe prop conditions. There was no statistical significant difference between toe prop conditions for mean peak apical pressure at the apex of the third fourth and fifth (sensor 2,3 and 4).

### Mean plantar digital pressure time integral

**Figure 6:**
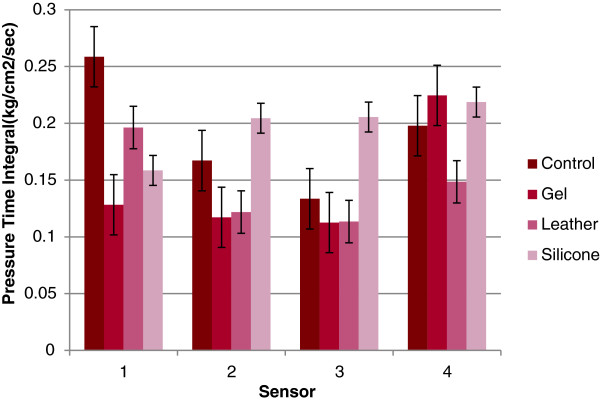
**Pressure time integral results.** Pressure Time Integral for each sensor for each condition with error bars.

The mean pressure time integral followed a similar trend to peak pressure with leather and gel toe props reducing the pressure time integral when compared to the control and silicone only achieving a reduction at apex of second digit (sensor 1) Figure 6. The results for apex 5^th^ digit (sensor 4) however, indicate an increase in the pressure time integral for the gel and silicone toe props. A significant difference was also observed in the mean pressure time integral at the apex of the 2^nd^ toe (sensor 1) (Wilks’ Lambda = .32, *F* (3,19) = 13.48, *p* < 0.001, multivariate partial eta squared = .68). The significance differences were seen whilst introducing the gel and silicone toe props. Again, there was no significant difference between conditions for mean pressure time integral at the apex of the third fourth and fifth (sensor 2,3 and 4).

### Comfort index

**Figure 7:**
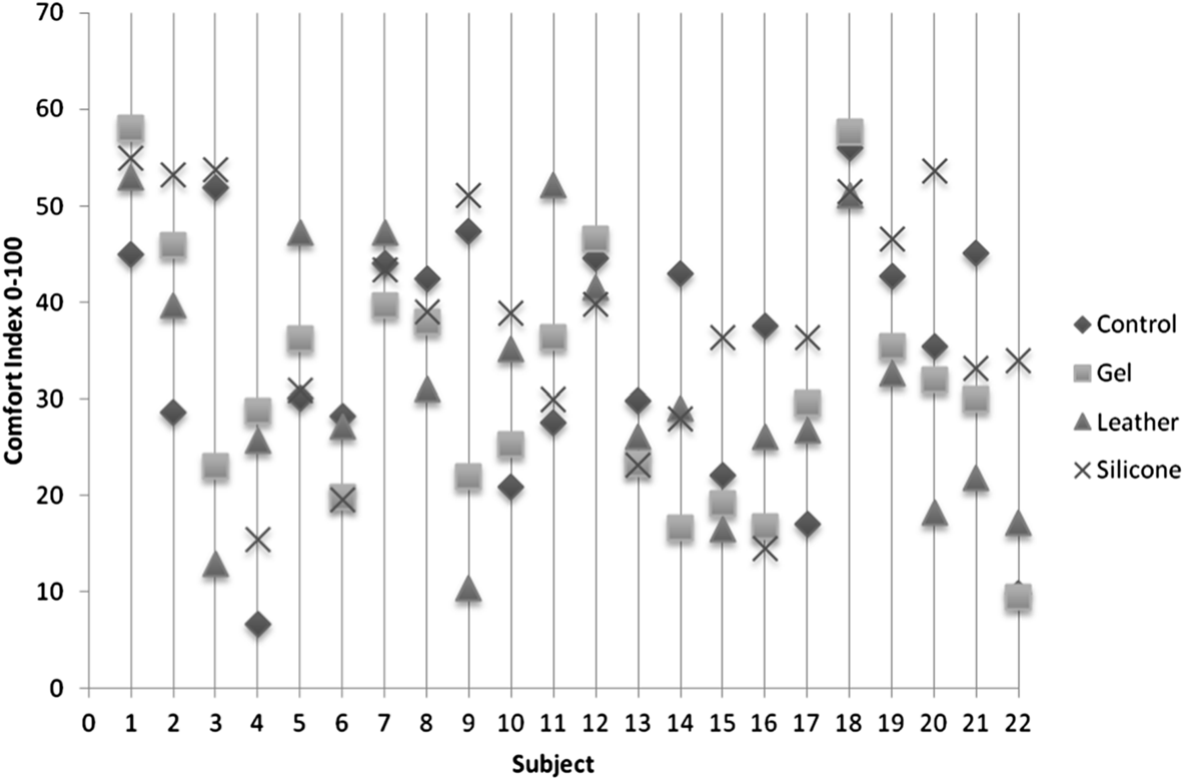
**Comfort Scores.** Comfort scores for each subject for each condition. The smaller the score the less comfortable the prop the higher score favours more comfort.

The scatter graph Figure 7 highlights the lack of relationship for the comfort of each condition. Each subject indicated different comfort scores for each of the toe props tested. There was little or no trend to be observed between conditions. It was found that 32% (n = 7) of subjects using a toe prop were more comfortable when compared to the control condition, with no specific trend between which toe prop was most comfortable. Similarly, 18% (n = 4) found the control condition more comfortable than any of the toe prop conditions. The statistical analysis using the Friedman test indicated there was no significant difference in the comfort scores across the four variables measured (*p* > 0.536. *χ*^2^ (3,n = 22) = 2.18, *p* < .0.005). Median values between control (*Md* = 36.5) and gel (*Md* = 29.8) leather (*Md* = 28.1) decreased. There was an increase in values for silicone (*Md* = 37.6) suggesting there may be a relationship in comfort for silicone toe props.

### Correlation between comfort and peak pressure

When comparing the recorded mean peak plantar digital pressure for the apex of the second digit (sensor 1) with the reported comfort scores for each variable there was no relationship observed. A Spearman rho correlation coefficient between mean peak plantar digital pressure and comfort for no toe prop *r* = 0.131, gel *r* = 0.74, leather *r* = 0.158 and silicone *r* = 0.15 showing no correlation between mean peak plantar digital pressure and comfort.

## Discussion

The results from this study highlight a significant reduction in peak plantar digital pressure and plantar digital pressure time integral on the apex of the second digit for the gel and silicone toe prop conditions. This demonstrates that the use of silicone and gel toe props are the most suitable current treatments when the desired objective is to reduce peak plantar digital pressure as well as pressure time integral for lesions on the apex on the second digit.

Lesion formation on the apex of digits has been associated to the amount of time pressure is raised in an area rather than the quantity of pressure [17,23]. A prolonged period of time under pressure deforms skin cells and instigates a hyperkeratotic response leading to the formation of callus and corns. If the lesion is not removed and the pressure continues the cells deform further breaking the epidermis and ulcerating the skin [12,13]. By reducing the peak pressure and pressure time integral this mechanism can be resolved and the patient’s symptoms are relieved. The three toe props investigated in this study are regularly used in clinical practice with understanding based on traditional concepts rather than research. Often toe props are dispensed on patient preference and past experience rather than clinical measures of reduced plantar digital pressure. This study has provided additional knowledge as to the effectiveness of toe props utilised in practice.

Although plantar digital pressure was reduced with silicone and gel toe props, patient compliance and comfort are often more important to the success of the treatment. Poor compliance often leads to the perception of the treatment being ineffective. Foot orthoses have been shown to be worn more when they are perceived as comfortable, delivering positive outcome measures to the success of the treatment [24]. Therefore making an effective treatment comfortable will enhance patient compliance and the outcomes achieved. This study highlighted no significant correlation between perceived comfort and reduction in pressure therefore it is not advisable to evaluate the impact of pressure reduction by the reported comfort level of the device. However it can be accepted that patients will not tolerate a toe prop device that is very effective in reducing pressure if it is not comfortable.

Furthermore, comfort analysis indicated that subjects found the silicone devices to be marginally more comfortable than the gel and leather toe props. With the knowledge that there was no significant change in peak pressure or pressure time integral when using a leather toe prop it could be further concluded that this prop has no clinical use for lesions on the 2^nd^ apex and should be replaced with a gel or silicone device that has been shown to reduce plantar digital pressure when treating apical lesions on the second digit.

The higher comfort index associated with silicone prop may also be in part due to the results of a higher contact surface area between the digits and the moulded silicone prop. The silicone prop covers the whole plantar surface of digits, whereas the gel and leather do not. The moulding technique, through which this kind of prop is manufactured, provides a bespoke contact surface area with the digits while complying with the shape of the lesser toes. This may increase the comfort associated with distributing the ground reaction force over a larger plantar surface and providing support in between the digits. Further studies are warranted to assess the effect of toe props on the plantar and digital pressure as well as interdigital. In such studies visco-elastic properties of the material which the prop is made off as well as the application technique that determined the geometry and contact area needs to be considered and scrutinised.

The population sampled in this study presented with a variety of toe deformities, all subjects had claw/hammer lesser toes with a prevalence of second toe deformities. Apical lesions were seen most frequently on the apex of the second digit which can bear 25% of digital peak plantar pressure and can be a main source of discomfort in the forefoot [1,9-11]. The success of the toe props in significantly reducing pressure in this area could be related to the type and frequency of this deformity within the population sampled. Although there were apical lesions present on the third digit also, this was not seen as a predominately deformed toe and could be associated with abnormal pressure at the second digit. Therefore, the distribution of second digital apex lesions within the sampled population may have promoted the significance of the results and further studies focusing on high frequency lesions on the third fourth and fifth apex are recommended.

## Conclusions

It can be concluded that the results of this study have demonstrated that there is a significant reduction in mean peak plantar digital pressure and pressure time integral on the apex of the second digit when using a gel and silicone toe prop. Furthermore from the sample studied the silicone toe prop was identified as more comfortable than the gel and leather toe props. It can therefore be recommended that the silicone toe prop is used as a primary treatment for second toe apical lesions associated with elevated peak plantar digital pressure and pressure time integrals.

## Competing interests

All authors involved in this manuscript can declare that they had no competing interests.

## Authors’ contributions

SJ and HB designed and conducted the study. SJ, HB and RN processed and analysed the data including statistical analysis. SJ, HB, NC produced and revised the manuscript. All authors reviewed the final manuscript before submission.
